# Sacred Birth, Clean Hands: Infection Prevention as Ritual and Practice among Traditional Birth Attendants in Mayuge District, East Central Uganda

**DOI:** 10.21203/rs.3.rs-6457438/v1

**Published:** 2025-04-17

**Authors:** Enid Kawala Kagoya, Proscovia Auma, Joshua Mugabi, Elizabeth Kawala, Deogratias Asabawebwa, Richard Mugahi, Paul Waako, Kenneth Mugabe, Banson Barugahare

**Affiliations:** Busitema University; Busitema University; Busitema University; Mayuge District Local Government; Ministry of Health; Busitema University; Busitema University; Busitema University; Busitema University

**Keywords:** Traditional Birth Attendants, Infection Prevention and Control, Maternal Health, Uganda, Ritual Hygiene, Hybrid Care Practices, Ethnography, Community Health, Safe Delivery, Cultural Beliefs

## Abstract

This study explored how Traditional Birth Attendants (TBAs) in rural Uganda implement infection prevention and control (IPC) through a blend of cultural traditions and hygiene practices. Using ethnographic methods, including interviews and observations, it found that TBAs view cleanliness as both a sacred obligation and a critical part of protecting maternal health. Despite facing limited training and resources, TBAs showed a strong commitment to infection prevention, positioning them as essential yet often under-recognized actors in maternal care.

**Introduction:**

The study investigated IPC practices among TBAs in rural Uganda, situating them within a broader cultural and spiritual framework. It emphasized how TBAs integrate traditional knowledge with selected biomedical practices to ensure maternal safety. This intersection of ritual and hygiene highlights the TBAs’ central role in rural maternal healthcare.

**Objective:**

The main objective was to explore how TBAs interpret, adapt, and implement IPC practices by drawing from both cultural beliefs and biomedical hygiene principles in the context of rural Uganda.

**Methods:**

A qualitative research approach was used, involving in-depth interviews and direct observations with 15 TBAs in Mayuge District. Data collection focused on everyday IPC practices such as waste disposal, hand hygiene, and use of protective materials. Thematic analysis helped identify recurring patterns and contextual challenges in IPC implementation.

**Results:**

The findings revealed that TBAs practice IPC through a hybrid model that blends ritual and hygiene. Placenta disposal, symbolic use of gloves, and improvised handwashing with soap, herbs, or oil were common. Despite limited formal training, TBAs showed innovation and commitment to safe deliveries, although resource scarcity and misalignment with formal guidelines posed challenges.

**Conclusion:**

The study highlighted the creative and spiritually informed IPC strategies of TBAs in rural Uganda, showing how they skillfully navigate material shortages and cultural expectations. These frontline caregivers operate with dedication and contextual intelligence, underscoring the need for culturally sensitive training and support to enhance maternal and newborn outcomes in under-resourced settings.

## Introduction

Infection prevention and control (IPC) are critical components of safe childbirth practices, especially in low-resource settings where access to formal healthcare services is limited[1]. Globally, maternal and neonatal infections remain significant contributors to mortality[2]. In 2023, approximately 260,000 women died from pregnancy-related causes, many of which were preventable[1]. Neonatal mortality also remains alarmingly high, with 2.3 million newborns dying within the first 28 days of life in 2022[1]. Saharan Africa bears a disproportionate burden of these deaths. In 2022, the region accounted for 57% of global under-five deaths, despite only representing 30% of global live births[1]. The neonatal mortality rate in sub-Saharan Africa was 27 deaths per 1,000 live births, the highest globally[2]. Eastern Africa, as part of this region, reflects similar challenges, with maternal and neonatal mortality rates remaining unacceptably high. Focusing on Uganda, the Busoga region exemplifies these challenges[2]. Busoga reported an institutional maternal mortality rate of 93 deaths per 100,000 institutional deliveries, exceeding the national average of 82.7[3]. Neonatal mortality in Busoga stood at 28 deaths per 1,000 live births, compared to the national rate of 22[1]. These statistics underscore the urgent need for effective IPC measures to safeguard maternal and neonatal health. Traditional Birth Attendants (TBAs) play a pivotal role in providing maternal care in rural Uganda, particularly in regions like Busoga[4]. Many women in these areas rely on TBAs due to factors such as cultural familiarity, accessibility, and trust. However, TBAs often operate with limited access to clean water, sanitation facilities, and medical supplies, raising concerns about infection prevention and control during childbirth[5].

Anthropological approaches offer valuable insights into how TBAs conceptualize cleanliness and infection. Rather than viewing IPC solely as a set of biomedical procedures, it is essential to understand how hygiene practices are interwoven with local beliefs, ritual purity, and moral obligations[6]. This perspective can inform culturally sensitive interventions that enhance IPC practices among TBAs[7]. The prevention of infections during childbirth involves several essential practices, including proper waste disposal, the use of personal protective equipment (PPE), and hand hygiene however, TBAs in rural areas face numerous challenges in maintaining these practices due to limited resources, insufficient access to hygiene materials, and a lack of formalized training[8]. Addressing these challenges requires a comprehensive understanding of the IPC methods employed by TBAs and the effectiveness of these practices in reducing maternal and neonatal infections[9]. By examining the IPC practices of TBAs, this research aims to offer insights into how traditional methods can be improved or integrated with modern healthcare practices to enhance the safety and well-being of mothers and newborns in rural settings. Such understanding is crucial for developing community-based solutions that work in tandem with formal health systems to improve maternal and child health outcomes.

## Objective

The objective of this study was to assess the IPC practices employed by TBAs in rural Uganda.

## Study site

Mayuge District is located in the southeastern region of Uganda, in the Eastern Central part of the country. It is bordered by Lake Victoria to the south, with other neighbouring districts including Jinja to the northwest, Iganga to the northeast, and Kamuli to the east. The district is part of the Busoga sub-region and is divided into both rural and urban areas, with Mayuge Town Council being the district headquarters. Mayuge District has a largely agrarian economy, with most of its population engaged in subsistence farming. The population of Mayuge District is predominantly rural, with a significant proportion of the population living in villages scattered throughout the district. Rural communities often face challenges such as limited access to healthcare, education, and infrastructure. Due to its rural nature, formal healthcare facilities in Mayuge District relies heavily on community-based healthcare services, including TBAs who provide maternal and neonatal care to expectant mothers. Access to skilled medical care, particularly in emergencies, is limited, and many women in rural areas continue to rely on TBAs.

## Methods

The study employed a qualitative research design, using in-depth interviews and direct observation of TBAs in rural Uganda. Data were collected from a sample of 15 TBAs in the Busoga region. Interviews focused on their infection prevention practices, including waste disposal techniques, the use of PPE, and hand hygiene. Observational data were also collected during home deliveries to assess real-time IPC practices. Thematic analysis was used to analyze the data, identifying common practices and challenges in IPC. This research employed qualitative ethnographic methods over a six-month period in 2024. The study was conducted in five villages in Mayuge District. Data collection involved: In-depth interviews with 15 TBAs Participant observation of birth-related rituals and hygiene practices in the homes and spaces where TBAs operate to explore collective narratives and shared practices around IPC. Interviews were conducted in Lusoga with the help of trained interpreters and transcribed verbatim. Thematic analysis was used to identify patterns related to beliefs, practices, and adaptations around infection prevention.

### Ethical Consideration

Ethical approval was sought and obtained from the Research Ethics Committees (REC) of Mbale Regional Referral Hospital No.MRRH-2023-342. Written informed consent was obtained from all subjects or participants and the consent form was approved by Mbale Regional Referral Hospital Research and Ethics Committee (REC) MRRH 2023 − 342.

## Results

### Social Demographic Factors

**Table:** 

Identifier	Age	Gender	Years of experience	Location
TBA1	45 years	Female	13 years	Buguwa
TBA2	34 years	Female	5 years	Nsaga
TBA3	52 years	Female	9 years	Nawanvubu
TBA4	38 years	Female	6 years	Waitambogwe
TBA5	54 years	Female	25 years	Buwalima
TBA6	60 years	Female	35 years	kigulu
TBA7	46 years	Male	20 years	Buwaiswa
TBA8	39 years	Female	15 years	Kikuubo
TBA9	44 years	Female	11 years	Mpungwe
TBA10	32 years	Female	9 years	Bukatuube
TBA11	38 years	Female	6 years	Mayuge
TBA12	39 years	Female	12 years	Nansaga
TBA13	42 years	Female	8 years	Bukatuube
TBA14	53 years	Female	16 years	Mayuge
TBA15	62 years	Female	30 years	Mpondwe

### Thematic Areas

The study explored various aspects of infection prevention and control (IPC) practices among Traditional Birth Attendants (TBAs) in rural Uganda. Qualitative research methods, including in-depth interviews and direct observations of home deliveries generated, key themes and subthemes. These themes provide insight into the practices and challenges TBAs face in maintaining infection control during childbirth.

**Table:** 

Main Themes	Sub-themes
**Waste Disposal Practices**	- Burial of contaminated materials
	- Use of placenta pits
	- Burning of contaminated materials
**Personal Protective Equipment (PPE)**	- Use of gloves and basic PPE materials
	- Availability and access to PPE
**Hand Hygiene Practices**	- Handwashing with soap or alternative materials (e.g., cooking oil)
	- Challenges with access to clean water
**Challenges in IPC Implementation**	- Limited access to hygiene materials and PPE
	- Lack of consistent training and resources
**Effectiveness of IPC Practices**	- Perceived effectiveness in reducing infections
	- Gaps in training and access to resources

### Theme 1: Waste Disposal Practices

Waste disposal is one of the most critical aspects of IPC during childbirth. Improper handling of biological waste such as placental material, contaminated clothing, and other medical waste can lead to infections and the spread of disease. The findings revealed several methods. TBAs used for waste disposal, each with its advantages and drawbacks. Although these waste disposal practices were culturally ingrained, they did not always align with modern IPC recommendations. Some of the disposal methods, such as burning, presented challenges in ensuring complete sterilization, while others, like burial, were dependent on the environment and did not always ensure safe waste disposal. TBAs expressed a desire for more education on proper disposal techniques, especially given the irregular access to hygiene materials.

#### Subtheme 1: Burial of Contaminated Materials:

Many TBAs in the Busoga region reported burying placental material and other contaminated items such as gloves and used bandages. Burial was seen as an effective method for preventing contamination in the immediate environment. However, this practice posed challenges, particularly when the burial site was not deep enough or located in close proximity to water sources, potentially leading to contamination of groundwater. Furthermore, in some cases, there was insufficient knowledge of hygienic burial practices.

“When we bury the placenta, it is not only for cleanliness but also to return it to the earth, to cleanse the area spiritually. It is a blessing to the earth, and this protects the mother and child (TBA4, Female, 38 years, Baitambogwe Village)”.

“While burial is symbolic, the lack of proper sanitation and the risk of contamination from shallow pits or poorly maintained disposal areas is a challenge. Sometimes we see mothers and babies falling ill after birth because of improper waste disposal (TBA1, Female, 45 years, Buguwa Village)”.

#### Subtheme 2: Use of Placenta Pits:

Some TBAs also employed placenta pits designated holes where placenta and other waste materials were disposed of. This method was deeply embedded in the cultural practices of the community and was considered an effective means of containing waste. However, a significant concern was that these pits were not always properly sealed or managed, which could lead to environmental contamination and increased risk of infection, particularly in areas prone to flooding or heavy rainfall.

“I have a placenta pit….. Points at it, it’s well constructed because when it rains, I don’t want to pollute the environment (TBA2, Female, 54 years, Bukatuube Village)”.

“My placenta pit is not that good, shows the team….. I hid it this far because what we are doing is illegal so if inspectors come, they will see it first and know that iam a TBA but I use it to dispose off all my waste (TBA3, Female, 52 years, Nawanvubu Village)”.

#### Subtheme 3: Burning of Contaminated Materials:

Burning of waste, including placental materials and soiled clothing, was another method reported by TBAs. While burning can effectively eliminate biological material, the lack of proper waste management infrastructure sometimes leads to incomplete combustion, leaving behind ash that could still harbour bacteria. Additionally, the act of burning in poorly ventilated areas posed health risks to the TBA, the mother, and the newborn.

“I first gather all the rubbish in that blue bucket and burn it over the weekend but in the night (TBA4, Female, 38 years, Baitambogwe Village)”.

### Theme 2: Personal Protective Equipment (PPE) Use

Personal protective equipment (PPE) is essential in preventing healthcare-associated infections. PPE minimize the exposure of healthcare providers and patients to blood-borne pathogens and other sources of infection. The use of PPE was inconsistent due to challenges of access and availability. While gloves were the most frequently used form of protection, improper handling, and reuse of gloves, as well as limited access to other PPE, posed significant risks to both the TBAs and their patients. The lack of access to PPE materials was a barrier to the consistent implementation of IPC guidelines.

#### Subtheme 1: Use of Gloves:

TBAs in the study commonly used gloves to handle contaminated materials during delivery. Gloves were used during procedures such as cutting the umbilical cord and handling the placenta. However, gloves were not always readily available, and TBAs often reused gloves if they had not been visibly soiled. This practice compromised their protective effect, as repeated use of gloves can lead to punctures or contamination of the exterior, potentially exposing the TBA to infectious material.

“We get gloves from the community pharmacies and drugshops and use them for some time (TBA15, Female, 62 years, Mayuge Village)”.

How do you do it?

“After attending to a patient, I sock them in water and wait for them to dry and use them on another patient because they are expensive yet patients give us little money. It’s not ideal, but we make it and If we had more support, we could do more to protect the mothers.(TBA9, Female, 44 years, Mpuugwe Village)”.

#### Subtheme 2: Black Plastic Bags:

Black plastic bags were sometimes used by TBAs to collect contaminated materials such as soiled cloths and tissues. These bags were not always sealed properly or disposed of immediately, leading to an increased risk of infection through improper handling.

“We use kaveera (plastic bags), you can take a look at one……brings it and shows….but these are also reusable, we wash them and use them on another patient (TBA11, Female, 38 years, Mayuge Village)”.

#### Subtheme 3: Limited access to PPE Materials:

One of the major challenges reported by TBAs was the inconsistent availability of PPE materials. TBAs often depended on informal networks to access gloves, aprons, and other protective materials. In some cases, TBAs resorted to using alternative materials such as pieces of cloth, which provided minimal protection.

“These PPEs are expensive out there, even some facilities don’t have, so when we don’t have, we use what is available (TBA5, Female, 54years, Buwalima Village)”.

Like what is available?

“She loughs…..i will never forget the day I used leaves to clean a mother, because she did not come with cotton then I wrapped the baby in my Gomesi…she loughs….(TB5, Female, 54 years, Buwalima Village)”.

### Theme 3: Hand Hygiene Practices

Hand hygiene is one of the most effective ways to prevent the transmission of infections during childbirth. The WHO recommends that healthcare workers should clean their hands thoroughly before and after patient contact to prevent the spread of harmful microorganisms. Hand hygiene was a priority for most TBAs, but the practice was undermined by limited access to clean water and soap. The use of cooking oil as an alternative to soap highlighted the resourcefulness of TBAs, but also underscored the limitations of their infection control practices in a context with insufficient hygiene materials. Efforts to improve access to clean water and soap are critical to enhancing hand hygiene practices in rural Uganda.

#### Subtheme 1: Use of Soap and Water:

Handwashing with soap and water was prioritized by most of the TBAs in the study. TBAs typically washed their hands before attending to the expectant mother, especially when preparing for the delivery. However, access to clean water and soap was often limited, particularly in more remote areas. In some cases, TBAs used makeshift alternatives like local plant-based soaps or ash, though these were less effective in killing harmful bacteria.

“Before attending to a mother, I wash my hands, not just for infection prevention but because it is a way of preparing myself spiritually. Clean hands are a sign of purity, and it is important to show respect to the mother and child (TBA6, Female, 60 years, Kigulu Village)”.

Despite the lack of soap and clean water in remote areas, TBAs creatively used alternatives like ash or herbal washes.

“If we don’t have soap, we use ash from the fire. It works well, and it is always available (TBA6, Female, 60 years, Kigulu Village)”.

“We also use herbs because they are local, and they come from our land (TBA10, Female, 32 years, Bukatuube Village)”.

#### Subtheme 2: Use of cooking oil as an alternative:

When soap and water were unavailable, some TBAs resorted to using cooking oil as an alternative for hand cleaning. While this practice was widespread, it raised concerns about its efficacy in removing pathogens. Cooking oil, although commonly used in rural settings, does not have the germicidal properties necessary for effective hand hygiene, and thus could fail to reduce the risk of infection.

“Sometimes we don’t have soap or water, so we use cooking oil to clean our hands. It’s available, and we believe it helps soften the skin, but it doesn’t kill the germs like soap would. I use it when doing an examination, especially if I’m feeling there is a lot of tension or discomfort for the mother (TBA7, Male, 44 years, Buwaiswa Village)”.

#### Subtheme 3: Challenges with Water Access:

A critical issue identified by the study was the lack of access to clean water. Many TBAs operated in areas with limited water sources, and some had to travel long distances to fetch water for handwashing. This hindered their ability to maintain consistent hand hygiene before and during childbirth, increasing the risk of infection.

“Water is very important, but we don’t always have it. Sometimes I have to travel far to fetch it, and when I return, I might be too late for the delivery. We do our best with what we have, but it’s difficult to maintain the kind of cleanliness we want(TBA12, Female, 39 years, Nansaga Village).”

“We know that washing hands is important, but there’s just not enough water around. During deliveries, I wash my hands, but it’s always a race against time. I use ash or leaves if there’s no soap, but water is the most important thing. Without it, I can’t be sure of preventing infection as I should (TBA8, Female, 39 years, Kikuubo Village)”.

### Theme 4: Challenges in IPC Implementation

Several challenges were identified that hindered the effective implementation of IPC practices among TBAs in rural Uganda. The lack of formal training in IPC, combined with limited access to hygiene materials and the influence of cultural practices, made it difficult for TBAs to consistently implement effective infection control measures. Providing training and better access to resources could help overcome these challenges and improve IPC practices in rural communities.

#### Subtheme 1: Limited Access to Hygiene Materials

The study revealed that TBAs often faced difficulties in accessing essential hygiene materials, including gloves, soap, and other forms of PPE. TBAs typically relied on informal networks or donations to obtain these items, and when they were unavailable, they resorted to alternative solutions that provided limited protection.

Sometimes we are told to use gloves and disinfectants, but where do we get them from? If no one brings them, we use what is available sometimes just a clean cloth or banana fiber. We can’t stop delivering babies because we don’t have gloves (TBA14, Female, 53 years, Mpungwe Village).

#### Subtheme 2: Lack of Formal Training

While TBAs had experience gained from years of assisting with deliveries, many had never received formal training in infection prevention and control. This lack of training resulted in a gap between traditional practices and modern IPC standards. TBAs were unaware of the full range of infection control techniques that could improve the safety of childbirth, such as sterilization of instruments or the proper disposal of waste.

“I learned from my grandmother and my aunties what they showed me is what I do. We were never taught by doctors or nurses about these things. We just do what we know keeps the mother safe. (TBA15,Female, 62 years old, Mayuge Village”

“Sometimes I hear the health workers say we should sterilize tools, but I don’t know what that means. I wash with water and dry them in the sun like my mother did

(TBA9, Male, 44 years old, Buwaiswa Village”

#### Subtheme 3: Cultural beliefs and practices

Cultural beliefs and practices also played a role in shaping the IPC methods used by TBAs. While some TBAs were open to adopting new techniques, others were more hesitant due to deeply ingrained traditional beliefs. These cultural practices sometimes conflicted with modern infection prevention methods, particularly when it came to waste disposal or the handling of placental material.

“We don’t throw the placenta just anywhere. If you do, bad spirits can follow the baby. That is why we bury it in a special place with herbs it protects both mother and child.”

(TBA3, 53 years old, Nawanvubu Village)

“The elders tell us that if you burn birth materials, you are inviting misfortune. That is why we must bury them properly, not burn them like the hospital people say.”

(TBA15, 62 years old, Mayuge Village)

### Theme 5: Effectiveness of IPC Practices in Preventing Infections

The effectiveness of IPC practices in preventing infections was a central concern of the study. Although TBAs were committed to infection prevention and had some basic IPC practices in place, the overall effectiveness of these practices was compromised by inconsistent access to resources and a lack of formal training. Integrating modern IPC guidelines with traditional methods and improving resource availability would enhance the effectiveness of infection prevention measures in rural Uganda.

#### Subtheme 1: Perceived Effectiveness

TBAs believed that their IPC practices, such as using gloves, washing hands, and disposing of waste, helped to reduce the risk of infections. However, many acknowledged that their practices were inconsistent due to the challenges of accessing necessary resources and materials. Despite these challenges, TBAs remained committed to ensuring safe deliveries to the best of their ability.

We believe that our practices using gloves, washing our hands with soap when we can, and disposing of waste properly are enough to ensure a safe delivery. We’ve been doing this for years, and we see the positive results (TBA1, Female, 45 years, Buguwa Village).

However, not all TBAs had access to the same resources or training. Another TBA added:

“Those TBAs who have been exposed to biomedical practices through training or community outreach have better infection control practices, but many still rely solely on traditional knowledge. This disparity can create uneven quality of care especially for us who haven’t benefited (TBA7, Female, 46years, Buwaiswa Village).”

#### Subtheme 2: Gaps in Training and Resources:

The study found that gaps in training and resources limited the overall effectiveness of IPC practices. TBAs who had received some form of informal training in IPC practices were more likely to implement effective measures, such as regular handwashing and proper use of gloves. However, those without such training often lacked the knowledge of more advanced infection control techniques, resulting in less effective practices.

We don’t have any formal training in infection control, but we’ve learned from experience and the advice of other elders. We do the best we can, but there are times when we wonder if we are doing enough (TBA13, Female, 42 years, Bukatuube Village).

## Discussion

The study revealed a range of IPC practices employed by TBAs in rural Uganda. The practices were often influenced by local traditions, limited resources, and a lack of formal training. TBAs employed various methods to dispose of contaminated materials, including burial, the use of placenta pits, and burning. These methods, although culturally accepted, varied in effectiveness. In some instances, these practices were inconsistent with modern IPC guidelines, as they did not eliminate contamination risks. While burial and burning were commonly used, there was a lack of awareness regarding more hygienic disposal methods as earlier reported [2]. The use of gloves and plastic bags, was common, however, access to these supplies was often irregular, and TBAs frequently had to rely on locally available materials like use of leaves and polythene. The shortage of appropriate PPE raised concerns about the potential for infection transmission. PPE is a fundamental component of infection prevention however, the lack of consistent access to these materials in rural Uganda could undermine efforts to control infections as outlined by WHO[10] Hand hygiene was prioritized by most TBAs, with regular handwashing before attending to expectant mothers however, access to soap and clean water was a significant challenge, particularly in remote areas. Some TBAs resorted to using alternative materials like cooking oil in the absence of soap, which raised concerns about the effectiveness of these practices in maintaining proper hygiene which was similar to the study conducted in Nigeria[11]. The study highlighted several challenges that hindered the effective implementation of IPC practices including limited access to hygiene materials, lack of formal training, and inconsistent availability of PPE not quite different from what was realized in Ethopia[12]. TBAs relied on traditional knowledge and practices, which were not always in line with modern infection prevention guidelines[13]. As a result, the effectiveness of IPC practices was compromised by these resource constraints, and the effectiveness of IPC practices was often seen as moderate but inconsistent. TBAs believed that their practices helped reduce the risk of infections, but the lack of resources and training limited the impact of these practices similar to what was found in Northern Uganda[4]. Integration of modern IPC practices with traditional methods could enhance the effectiveness of infection prevention efforts and improve maternal and newborn health outcomes. The findings highlight the importance of viewing IPC practices not just as technical interventions but as socially and culturally embedded practices. TBAs’ practices reflect a form of “pragmatic syncretism,” where spiritual rituals and biomedical techniques co-exist and inform each other. Cleanliness, in this context, is both a medical and a moral imperative. Understanding these hybrid practices provides possibilities for culturally sensitive health interventions just like the study in Nigeria suggested[11]. Rather than attempting to replace TBAs, health systems could engage them as partners, providing resources and training while respecting their local knowledge[14]. This approach can strengthen maternal health outcomes by building on existing trust and culturally resonant practices. This study highlighted the intricate and adaptive infection prevention and control (IPC) practices of Traditional Birth Attendants (TBAs) in rural Uganda, revealing a dynamic interplay between cultural traditions, spiritual values, and biomedical knowledge. Far from being passive custodians of ancestral wisdom[11], TBAs demonstrated a deep commitment to ensuring safe deliveries through creative, resourceful, and spiritually grounded practices[15]. Whether through the ritualized burial of waste, the symbolic and practical use of gloves, or alternative handwashing methods, TBAs operated as crucial maternal care providers in environments marked by material scarcity and infrastructural gaps. Despite facing significant challenges such as lack of formal IPC training, limited access to water and protective supplies, and tensions between traditional and hygiene paradigms similar to study done in Nigeria [16]. TBAs forged hybrid models of care that were both contextually meaningful and functionally effective not far from results of a study conducted in Bangladesh[17]. Recognizing and supporting these frontline caregivers with culturally sensitive training and sustainable resources is essential for improving maternal and newborn health outcomes in underserved regions.

## Conclusion

Traditional Birth Attendants in Mayuge District navigate infection prevention through a complex interplay of ritual, tradition, and hygiene practice paradigm. Their practices challenge narrow definitions of hygiene and invite a broader understanding of health that includes moral and spiritual dimensions. Recognizing and supporting this hybrid model of care can enhance both safety and cultural relevance in maternal health services.

## Data Availability

Transcripts used and/or analyzed during the current study are available from the corresponding author upon reasonable request.
